# Dispatches from the Interface of Salivary Bioscience and Neonatal Research

**DOI:** 10.3389/fendo.2014.00025

**Published:** 2014-03-04

**Authors:** Kristin M. Voegtline, Douglas A. Granger

**Affiliations:** ^1^Bloomberg School of Public Health, Johns Hopkins University, Baltimore, MD, USA; ^2^Institute for Interdisciplinary Salivary Bioscience Research, Arizona State University, Tempe, AZ, USA; ^3^Johns Hopkins University School of Nursing, Baltimore, MD, USA

**Keywords:** saliva analytes, saliva collection, testosterone, DHEA, estrogen, neonatal, analytical strategy

## Abstract

The emergence of the interdisciplinary field of salivary bioscience has created opportunity for neonatal researchers to measure multiple components of biological systems non-invasively in oral fluids. The implications are profound and potentially high impact. From a single oral fluid specimen, information can be obtained about a vast array of biological systems (e.g., endocrine, immune, autonomic nervous system) and the genetic polymorphisms related to individual differences in their function. The purpose of this review is to describe the state of the art for investigators interested in integrating these unique measurement tools into the current and next generation of research on gonadal steroid exposure during the prenatal and neonatal developmental periods.

## Introduction

The direction of research in the field of human development is being influenced by theoretical models that champion the study of individual differences using multi-level analyses of the confluence of prenatal, biological, behavioral, and social contextual forces [e.g., Ref. (1–4)]. Only recently have advances in salivary bioscience enabled investigators to test some of the core theoretical assumptions directly. Technical innovations now reveal that information may be obtained from oral fluid specimens about a broad array of physiologic systems including adrenal and gonadal steroids, inflammatory proteins of various types, infectious disease antibodies, environmental chemical exposures, metabolic markers, and genetic variability relevant to basic biological function, health, and disease. The attention that saliva has received as a biospecimen is largely due to the perceptions of sample collection as quick, uncomplicated, cost-efficient, and safe, and of salivary assays as reliable and accurate (see Table 1). The purpose of this report is to provide a roadmap for investigators interested in pushing cutting-edge integration of these measurement tools into the next generation of studies on gonadal steroid exposure and child development.

**Table 1:** **Perceived “advantages” of oral fluids as a research specimen**.

“Minimally invasive”	Considered “acceptable and non-invasive” by research participants and patients
	Collection is quick, non-painful, uncomplicated
“Safety”	Reduces transmission of infectious disease by eliminating the potential for accidental needle sticks
	CDC does not consider saliva a class II biohazard unless visibly contaminated with blood
“Self-collection”	Allows for community- and home-based collection
	Enables specimen collection in special populations
“Economics”	Eliminates the need for a health care intermediary (e.g., phlebotomist, nurse)
	Resources for collection and processing samples are low cost and available
“Accuracy”	Salivary levels of many analytes represent the “free unbound fraction” or biological active fraction in the general circulation

To date, the range of salivary analytes that have been integrated into developmental science has been restricted relative to the possibilities. The majority of studies with infants and children have primarily targeted cortisol as the salivary marker of choice given known associations with prenatal exposure [e.g., Ref. (5)], child behavior [e.g., Ref. (6, 7)], and in following with developmental origins theory, that early calibration of stress systems impacts health and development across the life course (8). However, the salivary proteome contains more than 1000 detectable analytes (9), including more understudied outputs of the hypothalamic–pituitary–gonadal (HPG) axis. Saliva contains measurable estrogens, androgens, and progesterone, yet few studies have collected oral fluid for this purpose in women during the antepartum and postpartum periods or in neonates (see Table 2); most have relied on serum.

**Table 2:** **Gonadal steroid salivary analytes of interest to developmental science and past human studies utilizing salivary assessment in the prenatal and neonatal periods**.

	Study population			Reference
	Antepartum women	Postpartum women	Neonates	
**ESTROGENS**				
Estradiol	x	x		(10–12)
Esterone	x	x		(10–12)
Estriol	x	x		(10–20)
**ANDROGENS**				
Testosterone	x	x	x	(11, 12, 21)
Dehydroepiandrosterone and -sulfate	x	x		(12)
Androstenedione^a^				
**PROGESTAGENS**				
Progesterone; 17-OH progesterone	x	x	x	(10, 12, 15, 16, 20, 22–24)
Aldosterone	x			(25)

*Sources to generate list of gonadal steroid analytes: Tabak (26); Malamud and Tabak (27); Cone and Huestis (28); U.S. Department of Health and Human Services (29)*.

*^a^To our knowledge, past studies have exclusively sampled plasma androstenedione; no studies to date have indexed salivary androstenedione in women during the antepartum or postpartum periods or in neonates*.

The extant literature on gonadal steroids focuses largely on levels observed during puberty and adulthood [see reviews (30, 31)]; we know far less about gonadal steroid exposure in early life and the majority of studies have relied on quantification from serum or plasma. However, not only do gonadal steroids of maternal origin cross the placental barrier and target the developing fetal brain (32, 33), but also birth marks a significant hormonal transition as the influence of the maternal endocrine milieu is withdrawn. In the first month following birth, infants exhibit a postnatal surge in gonadal steroids that parallel levels reached in adolescence, referred to as “mini-puberty” (34, 35). Further, steroid levels found in saliva, albeit lower in concentration, are correlated with serum constituents, particularly the free fraction which exerts physiological effects (12, 36).

To this end, we propose measurement considerations for oral fluid and applications of salivary analytes in studies of maternal and neonatal gonadal steroid activity for future investigation.

## Salivary Measurement Considerations

### Primer on oral fluid as a biospecimen

“Saliva” is a composite of oral fluids secreted from many different glands. The source glands are located in the upper posterior area of oral cavity, lower area of the mouth between the cheek and jaw, and under the tongue. There are also many minor secretory glands in the lip, cheek, tongue, and palate. A small fraction of oral fluid (crevicular fluid) also comes from serum leakage in the cleft area between each tooth and its surrounding gums, or via leakage from serum due to mucosal injury or inflammation. Under healthy conditions, the contribution from serum is minimal but under conditions of mucosal or epithelial inflammation, serum constituents can represent a substantial portion of the analytes in the oral fluid pool. Each secretory gland produces a fluid that differs in volume, composition, and constituents [e.g., Ref. (37)]. Each source gland’s contribution to the pool of oral fluid varies (38). Oral fluid is water-like in composition and has a pH (acidity) range between 6 and 9. Foods and substances placed in the mouth are capable of changing specimen acidity because oral fluid has minimal buffering capacity. These changes in sample integrity have been shown to compromise measurement validity [e.g., Ref. (39)].

Gonadal hormones are exemplars of salivary analytes that reflect levels in general circulation, otherwise known as serum constituents. Serum constituents are transported into saliva either by *filtration* between the tight spaces between cells in the salivary glands or *diffusion* through cell membranes thereby enabling investigators to make interferences about systemic physiological states. Other analytes found in oral fluids are synthesized, stored, and released from the granules within the secretory cells of the saliva glands (i.e., enzymes, mucins, cystatins, histatins). Still others are components of humoral (antibodies, complement) immunity or compounds (cytokines) secreted by cells (neutrophils, macrophages, lymphocytes) of the mucosal immune system. An understanding of whether an analyte is transported into oral fluid by filtration or passive diffusion, secreted from salivary glands, or released or derived from cells locally in the oral mucosa is key to interpreting the meaning of individual differences in that measure.

The secretion of oral fluids is influenced by the day–night cycle; chewing movement of the mandibles; taste and smell; iatrogenic effects of medications that cause xerostomia (dry mouth); as well as medical interventions and conditions that affect saliva gland function. Saliva glands are directly innervated by parasympathetic and sympathetic nerves (40). Not surprisingly, activation of the ANS component of the psychobiology of the stress response affects saliva flow rates. The levels of salivary analytes that migrate into saliva from blood by filtration through the junctions between cells in the salivary gland (e.g., dehydroepiandrosterone-sulfate and other conjugated steroids) are influenced by the rate of saliva secretion [e.g., Ref. (41)].

The U.S. Centers for Disease Control notes that unless visibly contaminated with blood, oral fluid is not a class II biohazard. This statement has contributed to the perception that, among behaviorally scientists, saliva is *safer* to work with than blood. In reality, even under normative-healthy conditions, more than 250 species of bacteria are present in oral fluids (42). During upper respiratory infections, oral fluids are highly likely to contain agents of disease (43). An informal survey of biosafety policies at North American and UK academic institutions reveals a consensus that oral fluid specimens should be handled with *universal precautions* when employed for use in research or diagnostic applications.

### Sample collection

In the past, saliva collection devices have involved cotton-based absorbent materials [e.g., Ref. (44, 45)]. Placed in the mouth for 2–3 min, oral fluids rapidly saturate the cotton; the specimen is expressed into collection vials by centrifugation or compression [e.g., Ref. (46)]. Most of the time, this approach is convenient, simple, and time-efficient. However, when the absorbent capacity is large and sample volume is small, as is the case for neonates, the specimen absorbed can be diffusely distributed in the cotton fibers, making sample recovery problematic [e.g., Ref. (47)]. The process of absorbing oral fluid with cotton, and other materials, also has the potential to interfere with the immunoassay of several salivary analytes (48, 49).

Early studies with neonates employed serum assays modified for use with saliva by, among other things, requiring large saliva test volumes (200–400 μl). To collect sufficient test volumes, saliva flow was often stimulated using techniques that involved tasting (sugar crystals, citric acid drops) substances. When not used minimally and/or consistently, some of these methods are capable of changing immunoassay performance [e.g., Ref. (39)]. Indirectly, stimulants also influence measurement of the levels of salivary analytes that are dependent on saliva flow rate (dehydroepiandrosterone-sulfate, DHEA-S). Saliva collected from neonates requires a technique that uses an absorbent material (50). Current collection methods use oral swabs, which are ideal for neonates and infants. This is because the swab can be narrower in diameter, particularly well-suited for the small mouths of neonates, and the material is non-toxic and durable for use with older infants that may gum the device during collection.

### Sample handling, transport, and storage

Typically, once specimens are collected, they should be kept cold or frozen. Refrigeration prevents degradation of some salivary analytes and restricts the activity of proteolytic enzymes and growth of bacteria. We explored the impact of bacteria-related issues on the measurement of salivary analytes. Significant declines occur in the levels of some salivary analytes when samples are stored at RT or 4°C in comparison to −60°C after 96 h (51). Whembolua and colleagues (52) showed that changes in salivary analytes related to storage temperatures were associated with growth in bacteria, but not baseline bacteria levels. The way in which samples are handled, stored, and transported after collection has the potential to influence sample integrity and measurement validity. Our recommendation is conservative. After collection, saliva samples should be kept frozen. If freezing is not possible, then at a minimum, samples should be kept cold (on ice or refrigerated) until they can be frozen that day. Repeated freeze–thaw cycles should be avoided with saliva samples. In our experience, DHEA, estradiol, and progesterone are very sensitive to freeze–thaw, whereas testosterone is robust (up to at least three cycles). This position is consistent with aliqouting and archiving frozen samples in anticipation that biotechnology advances will enable different markers to be assayed in the future. It should also be noted that some salivary analytes may require specimens to be directly collected into storage vials that are chilled [Ref. (53); but see Ref. (54)] or treated with neuropeptidase inhibitors (such as EDTA or aprotinin) to minimize rapid degradation (55, 56). For large-scale national surveys, investigators working in remote areas [e.g., Ref. (57, 58)], or patients collecting samples at home (59), freezing and shipping these frozen samples can be logistically complex and cost-prohibitive. Under special circumstances, saliva may not be the biospecimen of choice; alternatively, the degree of the impact of the handling and storage conditions should be documented by pilot work.

### Contaminants in oral fluid

Most of the time, to meaningfully index *systemic* biological activity, quantitative estimates of an analyte (e.g., hormone) in saliva must be highly correlated with the levels measured in serum. The magnitude of this serum–saliva association depends, in part, on consistency in the processes (27) used to transport circulating molecules into oral fluids. When the integrity of diffusion or filtration is compromised, the level of the serological marker in saliva will be affected because of the differential concentration gradient. It also depends on the presence of contaminants in oral fluid, which investigators should be aware of, control for if possible, and in all cases, document.

Blood and blood products can leak into oral fluids via burns, abrasions, or cuts to the cheek, tongue, or gums. Blood in oral fluid is more prevalent among individuals who suffer from poor oral health (i.e., open sores, periodontal disease, gingivitis), endure certain infectious diseases [e.g., HIV; Ref. (60)], and engage in behavior known to influence oral health negatively [e.g., tobacco use; Ref. (61)]. Saliva contaminated with blood will present varying degrees of yellow-brownish hue (62). Utilizing salivary transferrin as a surrogate marker, our bench (spiking blood into saliva) and experimental studies (inducing microinjury to the mouth by brushing the gums) have shown the degree of blood contamination needed to influence salivary hormone levels. Differences among the lengths of the effect (10–45 min) depend on the salivary analyte of interest (62–64). Gonadal steroids appear to be more susceptible to blood leakage in the oral cavity, with testosterone in particular showing an increase in response to microinjury in the oral cavity (62). At the same time, <0.1% of statistical outliers (+2.5 SDs) in salivary hormone distributions have been shown to be associated with elevated transferrin (65). Thus, blood contamination of oral fluid was a characteristic of individual specimens and not consistently detected within all samples from individuals. In studies of neonates and children, blood contamination is rare (65), however, studies of antepartum women who may present oral health risk should take heed to document and control for blood contamination.

A more universal concern, and one especially relevant in neonates, is contamination of samples from particulate matter and interfering substances placed in the mouth. Breastmilk, formula, and solids introduced later in infancy create residue in the oral cavity after drinking or eating may include particulate matter, change salivary pH or composition (viscosity), and/or contain substances (e.g., bovine hormones, enzymes) that cross-react in immune- or kinetic-reaction assays. Given the variation of infant feeding schedules and the difficulty in restricting intake, particularly when young infants are fed on demand, we recommend a simple solution: children should not be fed and adults should refrain from eating and drink with exception of water within the 20-min prior to sample donation. For repeated measure designs, saliva collection should be carefully planned and scheduled, and prior feedings noted.

Prescription and over-the-counter medications are also known contaminants to oral fluid, including those that are applied intranasally, inhaled or applied as oral topicals (e.g., teething gels). These substances have the potential to change saliva composition due to residue left by their use in the oral cavity, cross-reaction with antibody–antigen binding in immunoassays, and reduced salivary flow rate (66, 67). Confounding the effort to control for the potential effects of medications on salivary analytes is the possibility that the condition for which the medication is prescribed or taken may also influence individual differences in the analytes levels or activity (68). Few behaviorally oriented studies that involve salivary analytes have comprehensively documented medication usage. In one study of infants, nearly half (44%; *n* = 852) of the 6-month-old infants were given acetaminophen in the 48 h prior to saliva collection. Acetaminophen was shown to attenuate cortisol reactivity (69); implications for gonadal steroids remain unknown.

The lack of normative data and the wide-ranging individual differences in salivary analytes levels make case-by-case exclusion of samples from individuals taking any particular medication questionable (unless the deviation is obviously not physiologically plausible). Of course, the simplest approach would be to exclude anyone who is taking *any* medication from participation in research. This includes sampling from neonates of breastfeeding mothers who are themselves on a medication. Although appropriate from the perspective of rigorous experimental design, this conservative approach yields no information that may help us develop our knowledge of which medications are, or are not, problematic. Also, in studies of some specialized populations (e.g., pregnant patients with physical or mental illnesses), prescription medication use is so highly prevalent that it is considered normative. Withholding treatment raises ethical questions while excluding those taking medications increases the potential that findings may be confounded by selection bias. Future work is encouraged to document participant medication use and evaluate associations with steroid levels measured in saliva to build knowledge and document pharmaceutical contamination effects or lack thereof.

## Applications of Salivary Analytes in Studies of Maternal and Neonatal Gonadal Steroid Activity

Dependent on the nature of the research question, several sampling schemes are common for studies involving oral fluid. At the individual level, a single sample may be collected to index basal level, or multiple samples may be collected across the course of the day to capture diurnal rhythm. Concurrent sampling of a dyad is an additional scheme designed to evaluate associative relations over time. In further discussion of each sampling scheme below, we have embedded existing research drawn from Table 2 utilizing saliva to index maternal and neonatal gonadal steroid levels to encourage additional reading. Existing studies are relatively scant, yet saliva offers a wide array of application as a minimally invasive, accurate, and cost-effective approach; we offer suggestions for areas of future investigation with salivary analytes.

### Basal or “trait-like” levels

The basal level or activity of an analyte represents the stable state of the host during a resting period. One approach to assessing basal levels has been to sample early in the morning before the events of the day are able to contribute variation or specify a restricted time window for collection to reduce variation between participants [e.g., ±1200 h, Ref. (12)].

Single sampling is the most common approach for saliva collection, and the majority of studies referenced in Table 2 have employed this scheme. While overall the literature on salivary steroids during pregnancy and in the postpartum is lacking, studies examining estriol levels among antepartum women are most common (see Table 2). Estriol monitoring via saliva has been utilized as a clinical tool to assess the vitality of fetoplacental unit and a marker of preterm birth (17, 18). For this analyte, repeated measures of the “basal” level have been employed to detect atypical change [e.g., Ref. (18); weekly sampling from 22 weeks GA until birth]. As seen, the studies by Marrs and colleagues (11) as well as the more recent Hampson and colleagues (12) offer the most comprehensive report on gonadal steroids concentrations in antepartum and postpartum women and in comparison to serum, though limited to a single measurement during the third trimester of pregnancy. Future work is needed to elucidate reference values for gonadal steroids earlier in gestation, to evaluate normative endocrine changes of pregnancy and serve as a clinical tool for diagnosis of adrenal disorders during pregnancy.

We are only aware of a single study measuring neonatal salivary testosterone basal levels; findings revealed a positive association between salivary testosterone and neonatal health problems as well as growth delays among a high-risk preterm sample (21). A multitude of studies have explored neonatal cortisol reactivity, but more research is needed to understand the significance of the gonadal hormonal transition surrounding birth. Beyond the neonatal period, a handful of studies have sampled gonadal steroids to characterize “mini-puberty” observed during early infancy (70, 71). Testosterone levels of male infants peak between 1 and 3 months of age, and female infants show a spike between 2 and 4 months in follicle stimulating hormone (71, 72). The physiological relevance of this phenomenon, which exhibits universality across mammalian species, is theorized to activate sexual dimorphisms in the brain (72), remains underexplored to date. These analyses relied on single measures at varying ages to identify peak levels.

While a start, a single-time point measure of salivary analytes (other than invariant genetic polymorphisms), except under very unique circumstances, is *unlikely* to yield meaningful results for “basal levels” given that salivary analytes may vary depending on the inherent moment-to-moment, diurnal and/or monthly variation in their production/release, rate of their metabolism/degradation, and sensitivity to environmental influence. Given these issues, in combination with the moving target gonadal steroids present during the antepartum and neonatal/infant periods during which physiological shifts in hormone concentration are expected, cluster sampling for a series of three consecutive days is recommended. The association of salivary analyte measurements across days is likely to be stronger for some salivary analytes than others yet employing cluster sampling enhances overall reliability of the estimates by aggregating (e.g., average assay results or physically pool specimens). The minimally invasive nature of oral fluid collection is ideal for cluster sampling. Future studies are needed to describe the observed surge in gonadal steroids during infancy, investigate sex differences, and test predictive associations linking gonadal steroid activation to behavior.

### Diurnal rhythm

An important component of variability within and between individuals in salivary analyte levels is the diurnal rhythm of production [e.g., Ref. (73, 74)]. Most salivary hormone levels (e.g., cortisol, testosterone) are high in the morning, decline before noon, and then decline more slowly in the afternoon and evening hours (75). The non-linear nature of these patterns requires multiple sampling time points to create adequate statistical models (76).

The diurnal rhythm of gonadal steroid output in antepartum or postpartum women remains largely unexplored to date. There is evidence that the diurnal pattern is retained during pregnancy for other hormone outputs such as cortisol (77–79). For neonates, recent evidence suggests that in the first postnatal week the adrenal circadian rhythm becomes unsynchronized with clock time, peaking in late afternoon or at a time parallel to the birth time (80). The typical 24-h rhythm is generally not observed until after a few months for cortisol production, potentially coinciding with stabilization of the sleep–wake cycle. One study of salivary 17-OH progesterone indexed in neonates on the first or second day of life confirmed this, finding no evidence of a diurnal pattern (22). In a second cross sectional study designed to establish diurnal reference values for salivary progesterone and 17-OH progesterone, a diurnal pattern was observed among neonates <4 weeks age (*n* = 13, age range = 4–27 days) for 17-OH progesterone but not progesterone (23). Less is known about at what age gonadal steroids begin to show a characteristic diurnal rhythm; future studies documenting this would represent a considerable contribution to the literature, particularly during this rapid phase of development.

Analytical techniques that have been used to model individual differences in diurnal rhythm range from average levels, to focusing specifically on the awakening component of the overall pattern, to estimating AUC. Growth curve modeling (81) may be an optimal method of parameterizing individual differences because: (1) it allows the level and slope of the diurnal rhythm to be examined in the same model; (2) unsystematic error variance is partialed out of the “true” score; (3) the presence of individual differences in the diurnal rhythm is statistically tested; (4) predictors of the diurnal rhythm are related to level and slope in the same model (partialing out the effects of one another); and (5) changes in the variance estimates of the level and slope act as indicators of the overall contribution of the predictors [e.g., Ref. (81, 82)].

### Associative relations of salivary analytes between dyads

Physiological attunement or concordance of maternal and fetal hormones is documented before birth, *in utero*. For example, maternal prenatal testosterone serum levels are correlated with fetal levels ascertained from samples of amniotic fluid for both sexes (83). Further, women carrying male fetuses show higher testosterone concentrations relative to those carrying females (84) suggesting bidirectionality of observed associative relations.

Although recognized that in the postpartum period gonadal steroids may be decreasing in concentration among women while concurrently increasing among neonates, postnatal dyadic attunement of gonadal steroids remains an underexplored, yet promising, avenue for future research. The study noted in Table 2 that indexed neonatal salivary testosterone did find a positive correlation between testosterone levels in neonates and mothers in the first week after birth (21). Examining predictive associations from maternal levels during pregnancy to neonatal levels in the postpartum would also be of interest; for example, is there a greater or faster increase in gonadal steroid activity among neonates that experience more significant withdrawal from the maternal prenatal endocrine context (i.e., exposure to higher concentrations in the prenatal period).

The examination of associative relations need not be constrained to the dyad; at the individual level, this scheme carries over to studies exploring the coaction of two or more hormones. A single saliva sample of sufficient volume may be assayed for multiple analytes. For example, there is known functional cross-talk occurring between the HPG and HPA axes where gonadal steroids are postulated to induce a negative feedback loop in HPA reactivity (85). Gonadal steroids have also been theorized to stimulate oxytocin and in turn promote maternal behaviors in the postpartum period among mammals (86), as well as modulate immune and autoimmune response in general (87, 88). A dual systems approach that examines associative relations among two or more hormonal outputs (e.g., cortisol, oxytocin, SIgA) holds great promise for a deeper understanding of hormonal coaction during the antepartum and postpartum periods as well as calibration of early gonadal steroid activity in neonates and infants.

Typically, examination of relationships between two continuous variables involves the use of a summary statistic, such as a correlation; however, this is not always the most advantageous method. Nagin and Tremblay (89) used a model that jointly estimates growth mixture models for two distinct but related measurement series. The joint probabilities generated allow investigators to characterize the relationship between two dependent variables as they unfold over a specified period of time. Physiological attunement between members of the mother–child dyad appears to be a worthwhile application for this dual trajectory model.

## Conclusion

Parturition marks a host of physiological adaptations associated with changing environments; systems of respiration, circulation, homeostasis, and state regulation are just a few examples. While widely acknowledged that endocrine factors play an important role in this transition, the interrelationship of mother and infant gonadal steroids during this transition is not well understood. Beyond organizational effects, we know even less about how the transient activation of gonadal steroids in early postnatal life serves to differentiate child behavior and development.

As prenatal and neonatal gonadal steroid exposure can be reliably ascertained from saliva, and collected in a non-invasive manner particularly well-suited for research designs involving neonates and infants, oral fluid may become the biospecimen of choice for students of early human development. The purpose of this review was to provide a conceptual and tactical roadmap for investigators interested in integrating these measurement tools surrounding saliva into research on gonadal steroid exposure and health and human development. We continue to believe that the implications of the information divulged via this research will have a profound impact on developmental science in general, and the study of neonatal health and development in particular.

## Conflict of Interest Statement

The authors declare that the research was conducted in the absence of any commercial or financial relationships that could be construed as a potential conflict of interest.
